# Agricultural chemicals and gut microbiota dysbiosis: implications for the pathogenesis of inflammatory bowel disease

**DOI:** 10.3389/fnut.2026.1881234

**Published:** 2026-07-16

**Authors:** Guia Becherucci, Pierluigi Puca, Federica Di Vincenzo, Filippo Biamonte, Angelo Del Gaudio, Maria Chiara Mentella, Lucrezia Laterza, Pauline Raoul, Emanuele Rinninella, Daniele Napolitano, Alfredo Papa, Antonio Gasbarrini, Loris Riccardo Lopetuso, Franco Scaldaferri

**Affiliations:** 1 IBD unit – Digestive Disease Center (CeMAD), Fondazione Policlinico Universitario Agostino Gemelli IRCCS, Dipartimenti di Medicina e Chirurgia Traslazionale, Rome, Italy; 2 Clinical Nutrition Unit, Department of Medical and Abdominal Surgery and Endocrine-Metabolic Sciences, Fondazione Policlinico Universitario Agostino Gemelli IRCCS, Rome, Italy

**Keywords:** Crohn’s disease, dysbiosis, heavy metals, IBD, microbiota, mycotoxins, ulcerative colitis

## Abstract

The global intensification of agriculture has substantially increased human exposure to foodborne and environmental contaminants, including pesticides, heavy metals, endocrine-disrupting chemicals (EDCs), mycotoxins, and fertilizer-related related agricultural exposures. Growing evidence suggests that these agricultural xenobiotics may contribute to inflammatory bowel disease (IBD) pathogenesis through alterations of gut microbiota composition and function. This review examines the current evidence linking major agricultural contaminants to gut microbiota dysbiosis, intestinal barrier dysfunction, immune dysregulation, and chronic intestinal inflammation. Experimental and epidemiological studies indicate that chronic exposure to these compounds may reduce microbial diversity, alter the abundance of beneficial bacterial populations, impair short-chain fatty acid production, and activate oxidative stress and pro-inflammatory signaling pathways, including NF-κB and MAPK-related mechanisms. These alterations may collectively promote epithelial barrier disruption and aberrant mucosal immune activation, which are central processes in IBD pathogenesis. Attention is given to the role of dietary exposure and food-chain contamination as potential mediators of environmentally driven intestinal inflammation. Although most currently available evidence derives from *in vitro* and animal models, emerging human studies support a possible association between chronic exposure to agricultural contaminants and increased intestinal inflammatory risk. Overall, the evidence reviewed suggests that diverse agricultural contaminants, despite their different chemical characteristics, converge on common pathogenic mechanisms involving gut microbiota dysbiosis, oxidative stress, epithelial barrier dysfunction, and immune dysregulation. These shared alterations may represent a plausible mechanistic link between agricultural chemical exposure and intestinal inflammation, potentially contributing to increased susceptibility to IBD. However, the current body of evidence remains predominantly experimental, and causal relationships in humans have yet to be established. Future research should prioritize longitudinal human studies, exposome-based investigations, and integrated multi-omics approaches to clarify exposure–disease relationships, identify reliable biomarkers of exposure and risk, and support the development of preventive nutritional and environmental strategies targeting microbiota-mediated intestinal inflammation.

## 1 Introduction

**FIGURE 1:**
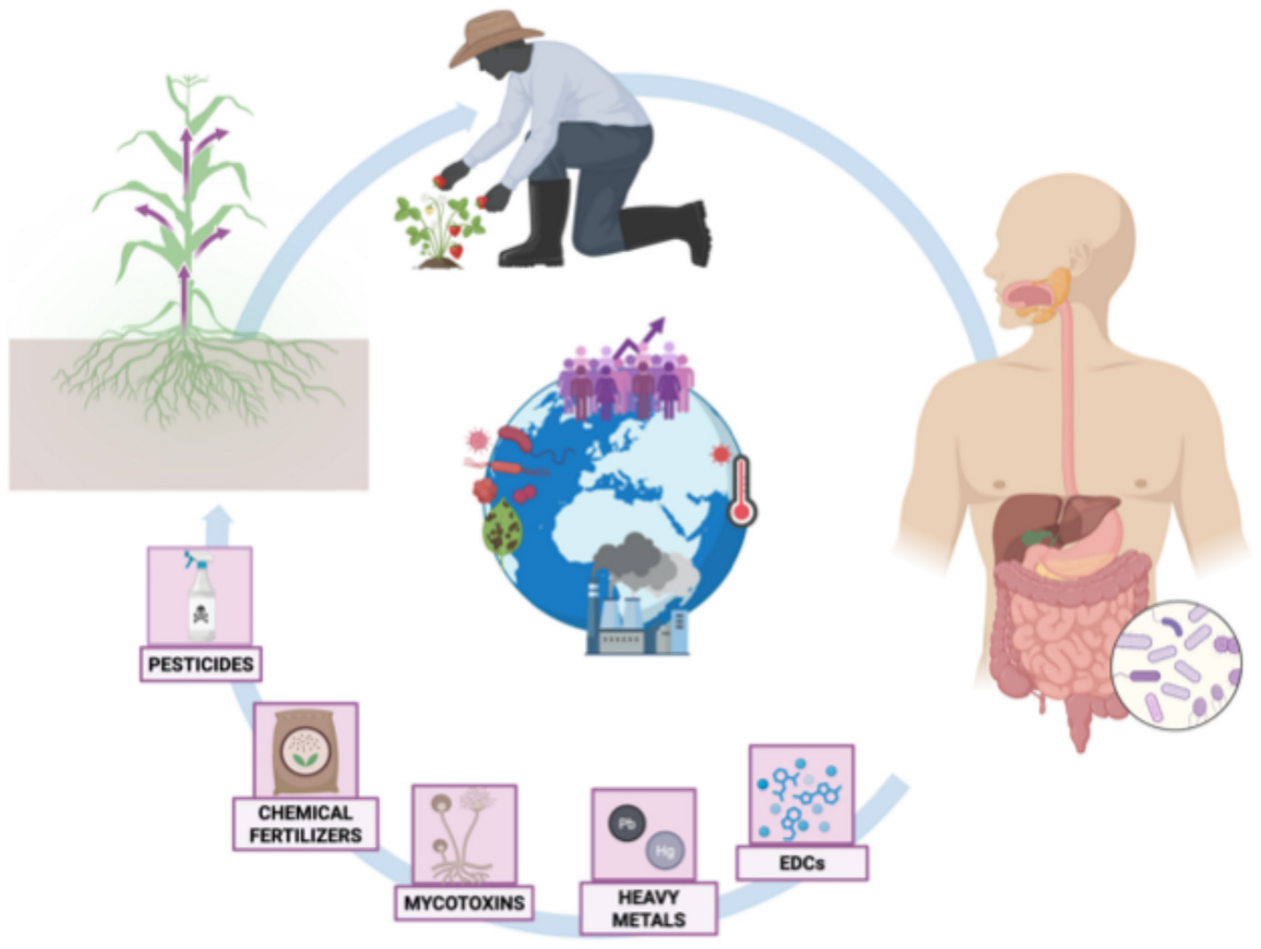
Pathway of agricultural chemicals from soil to human gut under environmental pressures.

Globally, the agricultural system is under increasing pressure to ensure sufficient, safe, and sustainable food production. According to the latest projections from the United Nations Department of Economic and Social Affairs, the world’s population will exceed 9.7 billion by 2050, leading to a significant increase in food demand (1). To cope with this growth, agricultural practices have progressively intensified, resulting in increased use of pesticides, fertilizers, and other chemical compounds (2). This scenario has led to a higher presence of environmental contaminants in food and environmental matrices, with potential implications for human health (3). Figure 1 summarizes the environmental transfer of agricultural contaminants to the human gastrointestinal system. Because diet represents the principal route of chronic exposure to many agricultural contaminants, nutritional habits and food-chain quality may play an important role in modulating gut microbiota alterations and intestinal inflammatory responses.

Chronic exposure to agricultural contaminants, including heavy metals, pesticide residues, mycotoxins, and endocrine disruptors, has been linked to the development of various chronic diseases (2, 4). In this context, the gut microbiota represents a crucial interface between environmental exposure and host health, as it is highly sensitive to exogenous factors and capable of modulating numerous physiological processes, including the immune response.

Inflammatory bowel diseases (IBD), including ulcerative colitis and Crohn’s disease, are conditions characterized by persistent inflammation of the gastrointestinal tract and a multifactorial etiology involving genetic, environmental, and immunological factors (5). Genetic variants affecting microbial sensing, autophagy, and host–microbe interactions, such as NOD2, ATG16L1, IRGM, and IL23R have been implicated in disease susceptibility, highlighting the complex interplay between host genetics and environmental influences (6). In this context, a key element in the pathogenesis of IBD is intestinal dysbiosis, defined as an alteration in the composition and function of the microbiota, leading to reduced microbial biodiversity, an expansion of pathobionts, and impairment of the intestinal barrier. These changes contribute to the aberrant activation of the immune system and the maintenance of a chronic inflammatory state (7, 8).

Evidence highlights how various classes of agricultural xenobiotics—including pesticides, heavy metals, endocrine disruptors, mycotoxins, and fertilizers—are widely distributed in the environment and characterized by significant persistence in biological systems, making them factors of toxicological interest for human health. These compounds are involved in various mechanisms, including oxidative stress, impairment of the intestinal barrier, and modulation of the immune response (7). In this context, the alteration of the gut microbiota induced by such exposures represents a possible key mechanism linking environmental contaminants to the development and progression of IBD (9–12).

Although accumulating evidence suggests that agricultural contaminants may contribute to gut microbiota dysbiosis and intestinal inflammation, most currently available mechanistic insights derive from experimental *in vitro* and animal models, whereas robust longitudinal human data remain limited. This review critically examines the impact of major agricultural contaminants on gut microbiota composition and function, with particular emphasis on their potential role in inflammatory bowel disease (IBD) pathogenesis.

## 2 Methodology

A narrative literature review was conducted using PubMed/MEDLINE, Scopus, and Web of Science databases to identify studies investigating the relationship between agricultural contaminants, gut microbiota alterations, intestinal inflammation, and inflammatory bowel disease (IBD). The literature search included articles published from database inception to January 2026 and used combinations of the following keywords: “gut microbiota,” “dysbiosis,” “inflammatory bowel disease,” “Crohn’s disease,” “ulcerative colitis,” “pesticides,” “heavy metals,” “endocrine-disrupting chemicals,” “mycotoxins,” “fertilizers,” and “agricultural contaminants.” Original research articles, experimental studies, epidemiological investigations, systematic reviews, and relevant review papers published in English were considered. Attention was given to studies exploring microbiota-mediated mechanisms potentially linking agricultural contaminant exposure to intestinal inflammation and IBD pathogenesis.

## 3 Pesticides

Pesticides represent one of the most extensively studied classes of xenobiotics, due to their widespread use and the substantial scientific evidence regarding their environmental persistence and potential effects on human health and ecosystems. As xenobiotics, they are chemicals of external origin capable of interacting with biological systems and accumulating in organisms if not properly metabolized and eliminated, causing alterations in fundamental physiological processes, such as hormonal regulation and cellular metabolism (10, 11).

To date, global pesticide use has been steadily increasing, primarily due to the need to ensure food security for a growing population and the intensification of agricultural practices, reaching approximately 4.3 million tons annually worldwide. This increasing use raises significant concerns regarding environmental and health effects, including loss of biodiversity, the development of pest resistance, and contamination of water resources (11, 13–15).

Gut microbiota plays a fundamental role in converting ingested compounds into bioactive metabolites which are essential for the proper functioning of the host’s nervous and immune systems and for maintaining immune balance, energy metabolism, and the integrity of the intestinal mucosal barrier (16–18). Chronic exposure to pesticides may alter gut microbiota composition (dysbiosis) and negatively affect immune balance at the gastrointestinal level (19, 20). Experimental studies have shown that exposure to individual pesticides or pesticide mixtures is associated with increased expression of genes involved in inflammatory pathways, including pro-inflammatory cytokines, leading to damage of the intestinal mucosa and colon (21).

Alterations in the gut microbial profile have been observed in animals exposed to different classes of pesticides, including organochlorines, organophosphates, fungicides, and carbamates. In particular, imbalances in major bacterial phyla, such as Firmicutes, Bacteroidetes, and Proteobacteria, together with reduced microbial biodiversity, have been reported (22, 23). However, the specific microbial alterations reported vary considerably according to the pesticide class, exposure conditions, and experimental model employed, making it difficult to identify a consistent microbiota signature associated with pesticide exposure.

Moreover, a recent study assessed the risk of IBD in individuals with high potential exposure to pesticides, highlighting an increased risk among those who lived in agricultural settings for at least 1 year during childhood or adolescence, particularly among those who applied them directly (24). Previous studies also suggest that pesticides can cross the placental barrier, with potential effects on the immune, endocrine, and metabolic systems during prenatal development (25, 26).

A further prospective cohort study in humans analyzed the relationship between exposure to specific pesticides and the onset of IBD, identifying an association between several organochlorine insecticides, including dieldrin, dichlorodiphenyltrichloroethane (DDT), and toxaphene, and an increased risk of IBD. The study also reported elevated hazard ratios for individuals exposed to organophosphate insecticides such as parathion, terbufos, and phorate, as well as to herbicides such as 2,4,5-T, 2,4,5-TP, and metolachlor, reinforcing the evidence of a positive association (27). Taken together, these associations may have been influenced by unmeasured factors including dietary habits, occupational exposures, smoking, and other lifestyle-related variables.

Pesticides can contribute to the inappropriate activation or suppression of the aryl hydrocarbon receptor (AhR). This receptor plays a key role in maintaining intestinal homeostasis by regulating epithelial integrity and the balance between regulatory and pro-inflammatory T cells. Its disruption can compromise intestinal immune function and promote conditions predisposing to the development of chronic inflammation (27, 28).

Among the different pesticide classes, herbicides have attracted particular attention because of their extensive worldwide use and growing toxicological relevance. Several experimental studies have investigated their potential effects on intestinal homeostasis and chronic inflammatory processes.

Glyphosate is one of the most widely used herbicides worldwide and one of the most frequently detected agricultural contaminants in environmental and biological matrices, including soil, water, food products, and human urine samples (29). Consequently, it has become a major focus of studies investigating the relationship between pesticide exposure, gut microbiota dysbiosis, and intestinal inflammation.

Experimental studies indicate that glyphosate exposure may directly impair intestinal epithelial integrity. In animal models, glyphosate administration reduced the expression of tight junction proteins, including zonula occludens-1 (ZO-1) in the jejunum and claudin-1 in the duodenum, suggesting increased intestinal permeability and disruption of mucosal barrier function (30).

Glyphosate-induced alterations in gut homeostasis also appear to involve oxidative stress pathways. In piglets, early-life exposure significantly increased catalase (CAT), superoxide dismutase (SOD), and malondialdehyde (MDA) levels in the duodenum, indicating activation of intestinal antioxidant responses and enhanced lipid peroxidation (30). Interestingly, these effects were region-specific and were not observed in the jejunum, suggesting differential susceptibility along the intestinal tract.

These findings are further supported by a study in young male rats exposed to glyphosate for 35 days, in which significant morphological alterations were observed throughout the intestine, including reduced villus height and a decreased villus-to-crypt ratio in the duodenum, jejunum, and ileum (31). These structural and molecular alterations were associated with activation of inflammatory and apoptotic pathways, including TNF-α, MAPK3, Caspase-3, and NF-κB signaling, which may collectively contribute to chronic mucosal inflammation and epithelial injury (32, 33).

However, although evidence supports a potential role of glyphosate in intestinal inflammation and disruption of gut homeostasis, their translational relevance to human health remains incompletely understood. Many experimental studies employ glyphosate doses ranging from tens to hundreds of mg/kg/day, substantially exceeding estimated human dietary exposure levels, which are generally below 0.01 mg/kg/day in the general population and remain under the current European acceptable daily intake (ADI) of 0.5 mg/kg body weight/day. Therefore, future studies should clarify whether chronic low-dose exposure, more representative of real-world human conditions, can induce intestinal and inflammatory alterations comparable to those observed in experimental models (34).

Diquat, another widely used bipyridyl herbicide, has also been associated with intestinal damage. Experimental studies indicate that it may intestinal mucosal integrity by reducing the expression of tight junction proteins, including occludin, claudin-1, and Zonula Occludens-1 (ZO-1) (35, 36).

Oxidative stress appears to be a key mechanism underlying these alterations. Animal studies have shown that Diquat promotes excessive production of reactive oxygen species (ROS), leading to oxidative damage of cellular membranes, proteins, and DNA in intestinal epithelial cells (37).

This oxidative imbalance may also induce mitochondrial dysfunction, thereby compromising epithelial barrier integrity and increasing intestinal permeability (38). Consistent with this mechanism, recent *in vitro* studies demonstrated that Diquat exposure enhances lipid peroxidation while reducing the activity of antioxidant enzymes, including superoxide dismutase and glutathione peroxidase. These alterations may activate pro-inflammatory pathways, leading to increased production of TNF-α, IFN-γ, IL-6, and IL-1β, as well as amplification of NF-κB signaling and intestinal inflammatory responses (39). The combined effects of oxidative stress, barrier dysfunction, and inflammation are accompanied by structural changes in the intestinal mucosa. Indeed, Diquat exposure has been associated with reduced villus height and increased crypt depth in animal models, suggesting impaired epithelial integrity and mucosal injury (40).

Experimental studies reported a reduction in potentially beneficial genera such as *Lactobacillus* and *Streptococcus*, together with a relative increase in members belonging to the phyla *Firmicutes* and *Actinobacteria*, as well as *Ruminococcaceae* and *Eubacterium coprostanoligenes* (39). These microbial alterations may further contribute to intestinal inflammation and oxidative stress, suggesting a bidirectional relationship between Diquat-induced dysbiosis and chronic intestinal damage (41).

Although Diquat has been banned in several countries, the intestinal toxic effects associated with its exposure continue to provide an important example of how environmental pollutants may disrupt intestinal homeostasis and contribute to inflammatory damage.

## 4 Heavy metals

Heavy metals such as cadmium (Cd), mercury (Hg), and lead (Pb) are persistent environmental contaminants known for their ability to bioaccumulate through the food chain and negatively impact human health.

### 4.1 Cadmium

Cadmium (Cd) is a persistent toxic heavy metal that primarily enters the food chain through the consumption of contaminated plants and food products. Although its gastrointestinal bioavailability in humans is relatively low (approximately 3–8%), chronic dietary exposure may lead to progressive bioaccumulation and long-term toxicological effects (42, 43). Increasing evidence suggests that cadmium exerts significant effects on gut microbial homeostasis and intestinal inflammatory responses through a complex bidirectional interaction with the gut microbiota.

On one hand, gut microbiota appears to modulate cadmium absorption, distribution, and excretion (43). Indeed, several studies have shown that probiotic administration, particularly involving *Lactobacillus* species, may reduce intestinal cadmium absorption and toxicity, suggesting a potential protective role of specific microbial populations (42, 43).

On the other hand, cadmium exposure may directly alter gut microbial composition. Experimental studies reported a reduction in beneficial bacterial families, including *Lachnospiraceae*, *Ruminococcaceae*, and *Prevotellaceae*, accompanied by decreased production of short-chain fatty acids (SCFAs) (43).

Cadmium-induced intestinal toxicity has also been associated with oxidative stress, mitochondrial dysfunction, and inflammatory signaling pathways (44), which, together with reduced SCFA production, may contribute to epithelial barrier impairment and intestinal inflammation, mechanisms potentially relevant in IBD pathogenesis (45).

Interestingly, a recent murine study highlighted the potential protective role of Rice Bran Insoluble Dietary Fiber (RBIDF) against cadmium-induced intestinal toxicity. RBIDF supplementation partially restored gut microbial balance, increasing the relative abundance of *Bacteroidetes* and *Lactobacillus* while reducing *Firmicutes* and *Proteobacteria* (46). These findings further support the concept that cadmium toxicity may be modulated by dietary factors and microbiota composition.

Nonetheless, evidence regarding cadmium-induced intestinal effects remains partly contradictory. In murine models of dextran sulfate sodium (DSS)-induced colitis (DSS) or 2,4,6-trinitrobenzene sulfonic acid (TNBS) induced colitis, short-term exposure to cadmium salts exacerbated disease severity, leading to increased weight loss and delayed recovery (47). By contrast, subchronic low-dose exposure to cadmium or lead through drinking water over 6 weeks (5 ppm Cd and 100 ppm Pb), concentrations selected to approximate environmentally relevant exposure conditions, produced limited effects in DSS-induced colitis and partially attenuated inflammatory responses in TNBS-induced colitis, including reduced histological damage and decreased expression of pro-inflammatory cytokines (48). In this context, it has been suggested that chronic cadmium exposure may induce adaptive responses within the gut microbiota characterized by patterns of resistance, adaptation, and sustained tolerance, which could contribute to the different inflammatory outcomes observed across experimental models (49).

Consequently, these apparently contrasting findings suggest that cadmium toxicity may largely depend on both exposure dose and duration. Acute and chronic cadmium exposure exert distinct effects on gut microbiota physiology and the fecal metabolome. While acute exposure is associated with rapid alterations in microbial function, prolonged exposure may lead to a progressive reorganization of host–microbiota interactions, thereby contributing to the different inflammatory outcomes observed in experimental models (48). These findings suggest that cadmium exposure may trigger dynamic host–microbiota adaptations capable of modifying immune responses over time, potentially explaining why both disease-promoting and partially protective effects have been reported under different experimental conditions.

### 4.2 Mercury

Human exposure to mercury, particularly methylmercury (MeHg), primarily occurs through the consumption of contaminated fish, seafood, wildlife, and, less frequently, certain pharmaceutical products. Due to its strong bioaccumulative properties and high affinity for biological tissues, methylmercury represents a major environmental toxicant with systemic inflammatory and neurotoxic potential (50). Experimental studies demonstrated that MeHg exposure induces a marked pro-inflammatory response in the colon. In murine models, methylmercury administration increased colonic levels of TNF-α and IL-1β, promoted neutrophil infiltration, and elevated fecal lactoferrin concentrations, indicating intestinal inflammation and mucosal immune activation (51). In parallel, increased oxidative stress markers were detected in colon tissues, suggesting that reactive oxygen species (ROS)-mediated toxicity may contribute to MeHg-induced intestinal injury. However, the translational relevance of these experimental findings should be interpreted cautiously, as some animal studies used acute high-dose exposure protocols rather than chronic low-dose exposure conditions. For example, in one murine study, HgCl2 was administered at 160 mg/L for 3 days, a dose selected on the basis of acute toxicity testing (1/20–1/5 of the LD50), rather than to reproduce realistic long-term human dietary exposure.

Mercury-induced toxicity is strongly associated with mitochondrial dysfunction and disruption of calcium homeostasis. In particular, MeHg can interfere with the mitochondrial electron transport chain, leading to excessive ROS production, oxidative stress amplification, and activation of inflammatory pathways (52). These mechanisms may contribute to chronic epithelial damage and sustained intestinal inflammation, processes implicated in inflammatory bowel disease (IBD) pathogenesis.

Although most evidence derives from experimental models, human exposure remains toxicologically relevant because of the strong bioaccumulative properties of methylmercury and the potential consequences of chronic dietary exposure, even at relatively low concentrations (53). Conversely, human toxicokinetic data based on EFSA-estimated dietary methylmercury intake support the relevance of chronic low-level exposure scenarios, showing that long-term dietary intake may contribute to sustained mercury accumulation in blood, hair, and target organs throughout life (53).

Supporting these experimental observations, epidemiological studies have also highlighted the systemic toxic effects of mercury exposure, reporting alterations in enzymatic activity and oxidative stress-related pathways in exposed individuals (54, 55). A recent prospective cohort study involving 22,824 participants followed for a mean duration of 5.24 years identified an association between long-term exposure to mercury and other environmental contaminants in drinking water and an increased risk of incident IBD (56). Notably, higher consumption of fruits and vegetables appeared to partially attenuate this risk, suggesting that dietary factors may modulate the inflammatory effects of environmental toxicants. Although the available findings are generally consistent, most evidence derives from experimental studies and the relevance of these mechanisms under chronic low-dose human exposure conditions remains to be clarified.

### 4.3 Lead

Lead (Pb) is a persistent environmental heavy metal widely distributed as a consequence of industrial and agricultural activities. Human exposure primarily occurs through contaminated soil, water, air, and food products, particularly in populations chronically consuming foods cultivated or raised in polluted environments (57). Due to its bioaccumulative properties and long biological persistence, lead exposure represents a relevant toxicological concern for intestinal and systemic health.

Experimental evidence indicates that lead exposure significantly alters gut microbiota composition and diversity, reducing overall microbial richness and disrupting the balance between aerobic and anaerobic bacterial populations. Alterations have been reported across multiple taxonomic levels, including changes involving the phyla *Firmicutes*, *Bacteroidetes*, and *Proteobacteria*, as well as bacterial families and genera such as *Lachnospiraceae*, *Erysipelotrichaceae*, *Desulfovibrionaceae*, *Streptococcaceae*, *Akkermansia*, *Desulfovibrio*, and *Parabacteroides* (58). Lead exposure appears to particularly affect mucus-associated bacteria, including *Akkermansia*, which play important roles in mucosal barrier maintenance and intestinal homeostasis. Although the specific microbial alterations reported vary across studies, disruption of bacteria involved in mucus layer maintenance and epithelial barrier integrity appears to be a recurrent finding.

Beyond microbiota alterations, several animal studies consistently reported impairment of intestinal barrier integrity following lead exposure, characterized by reduced mucus production and downregulation of key tight junction proteins, including ZO-1, occludin, and claudin-1 (59). In parallel, lead exposure modulates oxidative stress-related pathways through altered expression of antioxidant enzymes such as superoxide dismutase, glutathione peroxidase, and catalase (60). Prolonged exposure may also activate NF-κB signaling and promote the production of pro-inflammatory cytokines including IL-6 and TNF-α (61). Collectively, these alterations, together with gut microbiota dysbiosis, may contribute to epithelial barrier dysfunction and immune dysregulation and may represent mechanisms potentially relevant to IBD pathogenesis.

Despite substantial evidence supporting the pro-inflammatory effects of lead, some experimental colitis models reported reduced expression of specific inflammatory mediators following lead exposure, suggesting possible immunomodulatory or context-dependent effects (62). These apparently contradictory findings may reflect differences in exposure dose, exposure duration, and the experimental model used. Indeed, it has been observed that the immunological effects of lead are not linear and may vary according to exposure level, differentially influencing cytokine production and Th1/Th2 polarization. Moreover, the activation of antioxidant mechanisms in response to lead-induced oxidative stress may, in certain contexts, attenuate the expression of pro-inflammatory mediators such as IL-1β, TNF-α, and IL-6, thereby contributing to the variability of findings observed across experimental models. Beyond intestinal effects, chronic lead exposure is associated with significant systemic toxicity, particularly renal damage, neurotoxicity, and an increased risk of neoplastic diseases, including colorectal cancer (63, 64).

## 5 Endocrine-disrupting chemicals (EDCs)

Endocrine-disrupting chemicals (EDCs) are environmental compounds capable of interfering with hormonal signaling by altering hormone synthesis, release, transport, metabolism, and elimination. Increasing evidence suggests that EDC exposure may affect intestinal homeostasis by promoting immune dysregulation, epithelial barrier dysfunction, and gut microbiota alterations, mechanisms potentially involved in inflammatory bowel disease (IBD) pathogenesis (55, 65).

Among EDCs, bisphenol A (BPA) is one of the most extensively studied due to its widespread use in plastics, food containers, resins, and beverage packaging materials. BPA can migrate into food products, particularly under conditions involving heat exposure or contact with acidic and fatty foods (66). Its detection in multiple human tissues and biological fluids indicates pervasive environmental exposure and potential long-term accumulation (67). Human exposure to BPA generally occurs chronically at low doses through dietary intake and contact with food packaging materials, although internal exposure levels may vary according to dietary habits, age, occupational exposure, and environmental conditions. Importantly, several experimental studies have reported biological, inflammatory, and immunological effects even at relatively low exposure levels, including alterations in intestinal barrier integrity, oxidative stress responses, cytokine production, and immune homeostasis, raising concerns regarding the potential long-term impact of chronic BPA exposure on intestinal and systemic inflammatory processes. Notably, some experimental studies investigating BPA-induced intestinal alterations employed chronic exposure protocols at 50 μg/kg/day, a dose selected to approximate environmentally relevant exposure conditions and corresponding to the former EPA reference dose for BPA (68). Nevertheless, estimated human dietary exposure levels are generally lower, typically ranging from tens of ng/kg/day to low μg/kg/day, and this difference should be considered when interpreting the translational relevance of experimental findings (66, 67). Moreover, the recent EFSA re-evaluation substantially lowered the tolerable daily intake (TDI) for BPA, indicating that current human exposure levels may represent a potential health concern, particularly regarding immune and inflammatory effects (69).

Experimental evidence suggests that BPA may exert immunomodulatory effects, although the magnitude and direction of these responses appear to depend on dose, duration, and biological context. Studies have reported reduced T-helper cell proliferation and activity following BPA exposure, whereas combined exposure to multiple endocrine-disrupting chemicals (EDCs) may promote the expansion of regulatory T cells (Tregs), potentially contributing to immune dysregulation (70, 71). These apparently divergent findings likely reflect the complex and context-dependent immunomodulatory effects of BPA, which may differentially affect immune cell subsets and cytokine networks according to exposure conditions. However, because immune imbalance and altered T-cell responses play central roles in IBD pathogenesis (72), these findings suggest a possible mechanistic contribution of BPA to chronic intestinal inflammation. Consistent with this hypothesis, BPA exposure has also been associated with increased production of pro-inflammatory cytokines, including TNF-α, IL-6, IL-18, IL-23, and IL-17, supporting a role for BPA in the amplification of intestinal immune activation (68, 73). In both experimental and human studies, elevated cytokine levels following BPA exposure support the hypothesis that EDCs may contribute to intestinal immune activation and mucosal inflammation.

At the molecular level, BPA has been shown to modulate pathways involved in autophagy, cellular stress responses, and immune regulation. In particular, BPA may alter CALCOCO expression, a key regulator of mitophagy and cellular stress adaptation, and activate class A G protein-coupled receptor (GPCR) signaling pathways implicated in intestinal immune homeostasis (55, 74–76).

Beyond its immunological effects, it may also disrupt intestinal homeostasis through combined microbial, metabolic, and epithelial alterations. Experimental studies reported reduced production of short-chain fatty acids (SCFAs), particularly butyrate, disrupted tryptophan metabolism, and increased fecal calprotectin levels. In addition, BPA has also been associated with significant changes in gut microbiota composition, including the expansion of potentially pathogenic bacterial populations, particularly within the phylum *Pseudomonadota* (55, 71, 76, 77). In DSS-induced colitis models, these alterations were accompanied by increased disease severity and exacerbation of intestinal inflammation. Importantly, several studies also reported reduced expression of tight junction proteins, suggesting that BPA-induced microbial and metabolic disturbances may contribute to epithelial barrier dysfunction and increased intestinal permeability (73, 77, 78).

Human studies also support a possible association between BPA exposure and IBD-related inflammatory activity. A recent prospective observational study reported significantly higher serum BPA levels in patients with active IBD (11.98 ± 20.25 μM) compared with patients in remission (5.57 ± 8.29 μM). In particular, patients with active Crohn’s disease exhibited a positive correlation between serum BPA concentrations and fecal calprotectin levels, whereas patients with colonic involvement showed higher BPA levels compared to those with isolated ileal disease. Moreover, ileal tissue samples from patients with bacterial DNA translocation demonstrated markedly reduced mRNA expression of ZO-1 and claudin-1, suggesting impaired epithelial barrier integrity potentially associated with BPA exposure (73). However, the observational nature of these studies precludes causal inference, and the reported associations may be partially influenced by residual confounding and unmeasured environmental or lifestyle-related factors.

Despite increasing regulatory restrictions in several countries, including the recent European ban on BPA in food-contact materials (79), human exposure to BPA remains widespread globally, particularly because its use is still permitted in various industrial applications in countries such as the United States. Therefore, further longitudinal and mechanistic studies are needed to better define the translational relevance of current experimental findings.

## 6 Mycotoxins

Mycotoxins are a heterogeneous group of toxic secondary metabolites produced by filamentous fungi that frequently contaminate agricultural commodities and food products. Human exposure mainly occurs through the chronic consumption of contaminated cereals, grains, dairy products, meat, eggs, and other animal-derived foods originating from livestock fed contaminated feed (80). Due to their widespread occurrence within the food chain, mycotoxins represent a significant toxicological concern for intestinal and systemic health. Table 1 summarizes the principal food sources, predominant evidence, and major intestinal effects associated with the most relevant dietary mycotoxins discussed in this review.

**TABLE 1:** Main food sources and intestinal effects of major dietary mycotoxins.

Mycotoxin	Main food sources	Main intestinal effects	Predominant evidence	References
AFB1	Cereals, maize, nuts, dairy products	Immune dysregulation, lymphocyte alterations, dysbiosis	Mainly animal studies	(84, 87)
DON	Wheat, barley, maize, cereal-based foods	Barrier dysfunction, MAPK activation, reduced mucin production, inflammation	*In vitro* and animal studies	(83, 85)
OTA	Coffee, cereals, dried fruits, wine	Dysbiosis, oxidative stress, epithelial dysfunction	Animal but limited human studies	(84–86)
2′R-OTA	Roasted coffee	Long persistence in blood, slow elimination	Human clinical study	(82)

While the carcinogenic, mutagenic, and genotoxic properties of mycotoxins are well established, increasing attention has recently focused on their effects on gut microbiota composition, epithelial barrier integrity, and intestinal immune homeostasis (80, 81). Importantly, most current evidence regarding mycotoxin-induced intestinal toxicity derives from experimental *in vitro* and animal studies, whereas human translational data remain comparatively limited. Furthermore, experimental models generally investigate the effects of single mycotoxins under controlled conditions, whereas human exposure is characterized by chronic dietary co-exposure to multiple contaminants. This distinction should be taken into account when interpreting the translational relevance of current mechanistic evidence (80, 82, 83).

Experimental studies demonstrated that exposure to aflatoxin B1 (AFB1), deoxynivalenol (DON), and ochratoxin A (OTA) may induce significant gut microbiota alterations, including increased abundance of pro-inflammatory bacterial populations such as *Proteobacteria* and disruption of the *Firmicutes/Bacteroidetes* ratio (84–89). These dysbiotic changes have been associated with altered intestinal immune responses. In particular, DON and OTA exhibit strong pro-inflammatory properties by enhancing the expression of cytokines such as IL-1β and TNF-α (85, 86).

Mycotoxins may also affect lymphocyte populations involved in mucosal immune regulation. In animal models exposed to AFB1, reductions in CD4+ and CD8+ T-cell populations were observed within the intestinal mucosa, accompanied by structural alterations of lymphoid follicles in the lamina propria (84). As a consequence, evidence supports the concept that mycotoxins can simultaneously impair all major components of the intestinal barrier, including physical, chemical, immunological, and microbial defenses.

Among mycotoxins, deoxynivalenol (DON), primarily produced by species belonging to the *Fusarium graminearum* complex, represents one of the most extensively studied models of intestinal toxicity. Excessive DON exposure stimulates intestinal epithelial cells to release pro-inflammatory mediators, directly inducing mucositis and indirectly compromising mucosal barrier integrity (84).

DON also impairs goblet cell function by reducing mucin (MUC) production and suppressing the secretion of trefoil factor family (TFF) peptides, which are essential for epithelial repair and immune regulation. Consistent with these effects, experimental studies in human and porcine intestinal goblet cells exposed to controlled DON concentrations ranging from 0.5 to 16 μM demonstrated a significant reduction in TFF expression, suggesting impaired mucosal protection and epithelial healing (83). Although these controlled *in vitro* models provide important mechanistic insights, the concentrations employed may not fully reflect the complexity of chronic dietary exposure in humans, which generally occurs at lower doses and in combination with other mycotoxins (83). Moreover, through modulation of MAPK signaling pathways and related inflammatory mechanisms, DON promotes oxidative stress, epithelial dysfunction, gut microbiota alterations, and increased susceptibility to intestinal infections (90, 91).

Human exposure generally occurs chronically at relatively low dietary doses. A recent human clinical study evaluated the kinetics of 2′R-ochratoxin A (2′R-OTA), a thermal degradation product of ochratoxin A formed during coffee roasting. The study involved 16 healthy volunteers over 18 weeks and monitored blood and urinary toxin levels during periods of coffee consumption and abstinence. During the intervention phase, participants consumed coffee containing known concentrations of OTA (6.45 μg/kg) and 2′R-OTA (1.32 μg/kg). The results showed that 2′R-OTA persists for a long time in human blood, with an estimated biological half-life of more than seven months and very slow urinary elimination. The authors concluded that coffee represents the main source of human exposure to this mycotoxin and highlighted the need for further studies to clarify its potential toxicological effects and health risks in humans (82). Overall, current evidence suggests that chronic mycotoxin exposure may contribute to intestinal inflammation, epithelial barrier dysfunction, immune dysregulation, and gut microbiota alterations, which may potentially contribute to mechanisms involved in IBD pathogenesis.

## 7 Fertilizers

The use of fertilizers has become a fundamental component of modern agricultural productivity. Fertilizers are generally classified into two major categories: mineral (chemical) fertilizers and organic fertilizers. Mineral fertilizers, particularly those containing nitrogen, phosphorus, and potassium, are extensively employed to increase crop yields and improve agricultural efficiency.

However, intensive and prolonged application of mineral fertilizers may significantly alter soil microbial composition and reduce microbial biodiversity, potentially disrupting ecological processes such as nitrogen fixation, organic matter degradation, and carbon cycling (92). These alterations may indirectly affect crop nutritional composition, including reductions in dietary fiber, polyphenols, and essential micronutrients that are important for maintaining gut microbiota homeostasis.

In contrast, organic fertilization practices have been proposed as a potential strategy to support soil biodiversity and ecosystem functionality. They may enhance soil microbial diversity and influence crop composition, potentially supporting gut microbiota homeostasis through increased levels of bioactive compounds and food-associated microorganisms (93–96).

Despite these observations, direct evidence linking fertilizer-related agricultural practices to intestinal inflammation and inflammatory bowel disease (IBD) remains limited. Most available studies focus on ecological and nutritional outcomes rather than mechanisms directly involved in intestinal inflammation. Importantly, there is currently no direct evidence demonstrating that fertilizer residues themselves, at physiologically relevant concentrations, directly induce gut dysbiosis, intestinal inflammation, or IBD. Although organically produced foods may contain lower levels of contaminant residues and higher concentrations of certain bioactive compounds, clinical evidence supporting direct health benefits remains limited and inconsistent (97–102).

Therefore, the potential relationship between fertilizer-related agricultural practices, gut microbiota alterations, and intestinal health remains largely speculative and supported primarily by indirect evidence. Further mechanistic and clinical studies are required to determine whether these associations contribute to intestinal inflammation and IBD pathogenesis.

## 8 Future perspectives

The accelerating global demand for food is driving agriculture toward increasingly chemical-intensive practices, with growing use of pesticides, fertilizers, and other environmental contaminants. This trend raises important concerns regarding food quality, gut microbiota homeostasis, and the potential contribution of chronic environmental exposures to inflammatory bowel disease (IBD). Although accumulating evidence suggests that agricultural chemicals may impair intestinal barrier integrity, immune regulation, and microbial composition, the mechanistic basis of these interactions remains incompletely understood, particularly in humans. Importantly, the quantity and strength of the available evidence vary considerably across contaminant classes and according to the type of exposure and study design. A qualitative overview of the current evidence is summarized in Table 2.

**TABLE 2:** Summary of the current level of evidence supporting the association between major agricultural contaminant classes, gut microbiota alterations, intestinal inflammation, and IBD-related mechanisms.

Contaminant class	*In vitro* studies	Animal studies	Human observational studies
Pesticides	Extensive	Extensive	Moderate
Heavy metals	Extensive	Extensive	Moderate
EDCs	Extensive	Extensive	Limited–moderate
Mycotoxins	Moderate	Moderate–Extensive	Limited
Fertilizer-related agricultural practices	Limited	Limited	Indirect evidence

High-resolution multi-omics approaches, including metagenomics, metabolomics, transcriptomics, and exposomics, may help clarify how chronic exposure to agricultural chemicals reshapes microbial ecosystems, alters host metabolic pathways, and promotes early intestinal damage. Identification of these molecular signatures could improve risk prediction, biomarker discovery, and the development of preventive or therapeutic interventions targeting environmentally induced intestinal inflammation.

**FIGURE 2:**
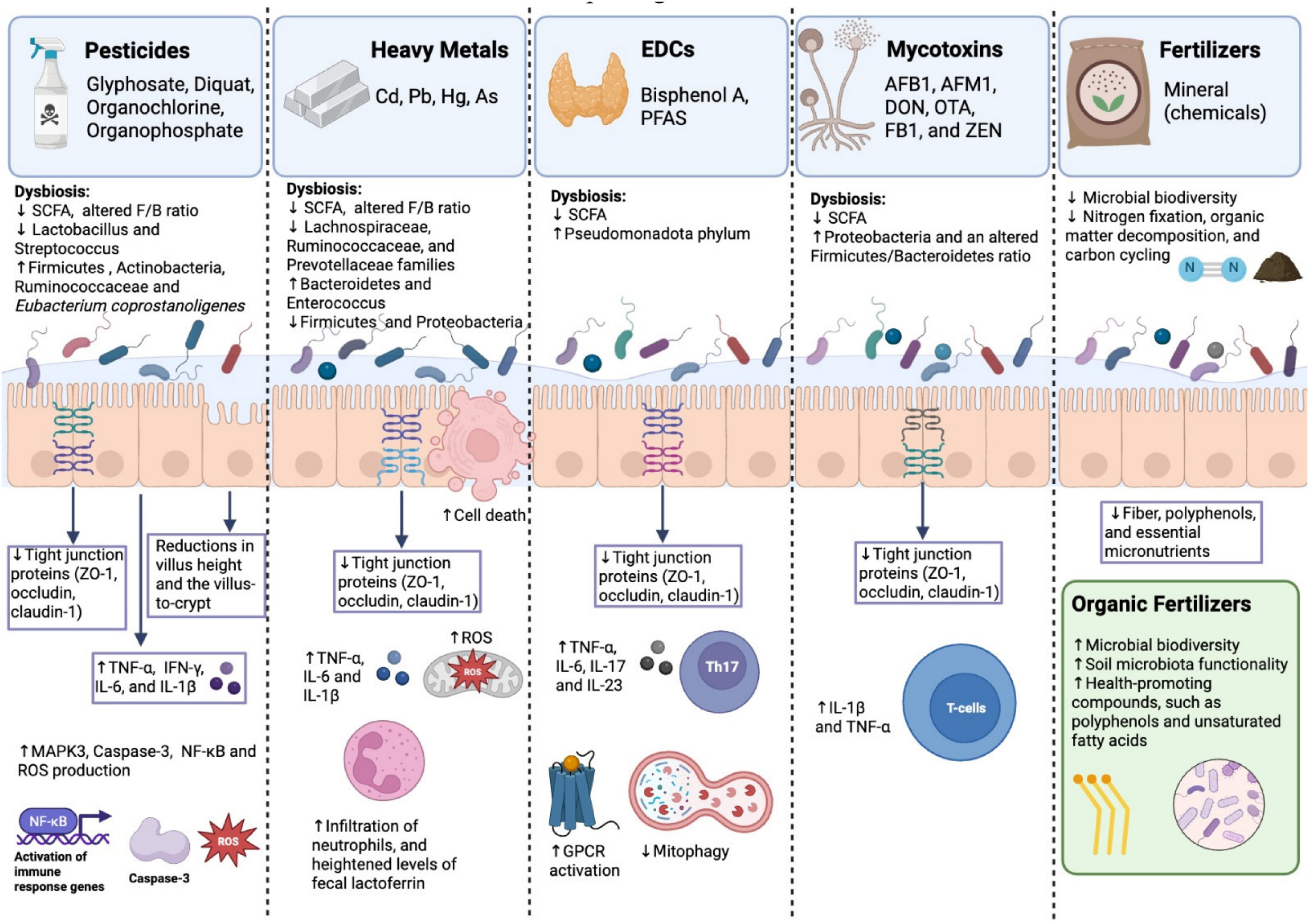
Proposed mechanisms linking agricultural xenobiotics to gut microbiota dysbiosis and intestinal inflammation in inflammatory bowel disease (IBD). Agricultural contaminants, including pesticides, heavy metals, endocrine-disrupting chemicals, mycotoxins, and fertilizer-related exposures, may alter gut microbial composition and metabolic activity, leading to reduced short-chain fatty acid (SCFA) production, oxidative stress, and epithelial barrier dysfunction. These alterations may promote increased intestinal permeability, activation of inflammatory signaling pathways (including NF-κB and MAPK), and aberrant mucosal immune responses, ultimately contributing to chronic intestinal inflammation. The arrows indicate the proposed directionality of these interactions. Continuous lines represent mechanisms supported by experimental evidence, whereas the overall integration of these pathways into IBD pathogenesis remains a proposed model based on currently available evidence. SCFA, short-chain fatty acids; ROS, reactive oxygen species; ZO-1, zonula occludens-1; NF-κB, nuclear factor kappa B; MAPK, mitogen-activated protein kinase; GPCR, G protein-coupled receptor.

Future studies should also better characterize the toxicological relevance of chronic low-dose exposure conditions that more closely reflect realistic human dietary and environmental exposure scenarios. Longitudinal and interventional human studies will be particularly important for distinguishing causal relationships from indirect or confounded epidemiological associations. Despite their different chemical structures and sources of exposure, many of the contaminants discussed in this review appear to converge on common biological pathways involved in intestinal homeostasis. Recurrent alterations in gut microbiota composition, epithelial barrier integrity, oxidative stress responses, and immune regulation were observed across pesticides, heavy metals, endocrine-disrupting chemicals, and mycotoxins. At the molecular level, activation of ROS-dependent pathways, NF-κB/MAPK signaling, cytokine dysregulation, and disruption of tight junction proteins emerged as potential mechanisms linking environmental exposures to intestinal inflammation. An overview of these interconnected mechanisms is presented in Figure 2.

In this context, sustainable agricultural systems, including organic and regenerative farming approaches, have attracted increasing scientific interest as potential strategies to reduce environmental contaminant exposure and improve food quality (102, 103). Regenerative agricultural practices, such as reduced tillage, cover cropping, and biologically integrated nutrient management, may contribute to restoring soil biodiversity and ecosystem resilience (103, 104). In addition, several studies and meta-analyses reported that organically produced foods may contain lower levels of pesticide residues and heavy metals, together with higher concentrations of bioactive compounds with antioxidant properties (99, 105, 106). However, current evidence linking regenerative agricultural practices to direct modulation of gut microbiota composition or improved IBD outcomes remains limited and largely indirect. Additional mechanistic and clinical studies are therefore required to validate these potential benefits and assess their long-term translational relevance.

Future research should also investigate whether nutritional interventions targeting gut microbiota composition, including high-fiber diets, polyphenol-rich foods, probiotics, prebiotics, and Mediterranean dietary patterns, may mitigate the intestinal effects associated with chronic exposure to agricultural contaminants. Dietary patterns rich in fiber, polyphenols, antioxidants, and fermented foods may help preserve microbial diversity, promote short-chain fatty acid production, and support epithelial barrier integrity. These nutritional approaches may therefore represent promising strategies to counteract microbiota dysbiosis and chronic intestinal inflammation.

Overall, growing evidence suggests that the global dependence on chemically intensive agriculture may have important implications for intestinal health and chronic inflammatory diseases. Future research integrating toxicology, microbiology, immunology, nutrition, and environmental sciences will be essential to better understand how agricultural practices influence gut health and to support the development of safer and more sustainable food systems.

## 9 Limitations

This review has several limitations. First of all, most currently available data derive from experimental animal models, *in vitro* systems, or observational human studies, frequently involving heterogeneous exposure conditions and limited translational applicability. In addition, many experimental studies employ exposure doses substantially higher than those encountered under realistic human dietary conditions. Human exposure also rarely occurs in isolation, and the combined effects of multiple contaminants remain difficult to disentangle from other factors that may influence gut microbiota composition and IBD risk. Indeed, observational studies may be affected by potential confounding variables, including dietary habits, smoking, antibiotic use, occupational exposures, lifestyle factors, genetic susceptibility, and host-specific microbiota variability. Causal relationships between agricultural chemicals, microbiota alterations, and IBD pathogenesis remain incompletely established and require further longitudinal and mechanistic investigation.

## 10 Conclusion

Agricultural chemical exposure may influence biological pathways involved in gut homeostasis and intestinal inflammation that are relevant to IBD pathogenesis. Current evidence suggests that pesticides, heavy metals, endocrine-disrupting chemicals, mycotoxins, and fertilizer-related agricultural practices may converge on common mechanisms, including gut dysbiosis, epithelial barrier dysfunction, oxidative stress, immune dysregulation, and activation of pro-inflammatory pathways. However, important translational limitations remain, and future research should prioritize longitudinal human studies and integrated multi-omics approaches. Understanding how foodborne environmental contaminants interact with gut microbiota and intestinal immunity may provide new opportunities for preventive nutritional strategies targeting environmentally driven chronic inflammation and IBD susceptibility.
